# Effects of body mass index on the immune response within the first days after major stroke in humans

**DOI:** 10.1186/s42466-023-00269-1

**Published:** 2023-08-17

**Authors:** Johanna Ruhnau, Christin Heuer, Carl Witt, Sonya Ceesay, Juliane Schulze, Stefan Gross, Maria Waize, Marie-Luise Kromrey, Jens-Peter Kühn, Sönke Langner, Uwe Grunwald, Barbara M. Bröker, Astrid Petersmann, Antje Steveling, Alexander Dressel, Antje Vogelgesang

**Affiliations:** 1 Department of Neurology, University Medicine Greifswald, F.-Sauerbruch-Str, 17475 Greifswald, Germany; 2 Partner site Greifswald, German Centre for Cardiovascular Research (DZHK), Greifswald, Germany; 3 Department of Mathematics and Informatics, University Medicine Greifswald, Greifswald, Germany; 4 Department of Department of Diagnostic Radiology and Neuroradiology, University Medicine Greifswald, Greifswald, Germany; 5 Institute and Policlinic of Diagnostic and Interventional Radiology, University Hospital Carl Gustav Carus, Dresden, Germany; 6 Department of Clinical Diagnostics, University Oldenburg, Oldenburg, Germany; 7 Department of Immunology, University Medicine Greifswald, Greifswald, Germany; 8 Department of Endocrinology, University Medicine Greifswald, Greifswald, Germany; 9 Department of Neurology, Carl-Thiem Klinikum, Cottbus, Germany; 10 Internal Medicine C, Hematology and Oncology, University Medicine Greifswald, Greifswald, Germany

**Keywords:** Obesity, Stroke, Poststroke immune modulation

## Abstract

**Introduction:**

Immunological alterations associated with increased susceptibility to infection are an essential aspect of stroke pathophysiology. Several immunological functions of adipose tissue are altered by obesity and are accompanied by chronic immune activation. The purpose of this study was to examine immune function (monocytes, granulocytes, cytokines) as a function of body mass index (BMI: 1st group: 25; 2nd group: 25 BMI 30; 3rd group: 30) and changes in body weight post stroke.

**Method:**

Fat status was assessed using standardized weight measurements on days 1, 2, 3, 4, 5, and 7 after ischemic stroke in a cohort of 40 stroke patients and 16 control patients. Liver fat and visceral fat were assessed by MRI on day 1 or 2 [I] and on day 5 or 7 [II]. Leukocyte subpopulations in peripheral blood, cytokines, chemokines, and adipokine concentrations in sera were quantified. In a second cohort (stroke and control group, n = 17), multiple regression analysis was used to identify correlations between BMI and monocyte and granulocyte subpopulations.

**Results:**

Weight and fat loss occurred from the day of admission to day 1 after stroke without further reduction in the postischemic course. No significant changes in liver or visceral fat were observed between MRI I and MRI II. BMI was inversely associated with IL-6 levels, while proinflammatory cytokines such as eotaxin, IFN-β, IFN -γ and TNF-α were upregulated when BMI increased. The numbers of anti-inflammatory CD14^+^CD16^+^ monocytes and CD16^+^CD62L^−^ granulocytes were reduced in patients with higher BMI values, while that of proinflammatory CD16^dim^CD62L^+^ granulocytes was increased.

**Conclusion:**

A small weight loss in stroke patients was detectable. The data demonstrate a positive correlation between BMI and a proinflammatory poststroke immune response. This provides a potential link to how obesity may affect the clinical outcome of stroke patients.

**Supplementary Information:**

The online version contains supplementary material available at 10.1186/s42466-023-00269-1.

## Introduction

Stroke, a major cause of disability and death worldwide, is associated with obesity mostly measured by body mass index (BMI). Obesity is usually defined as the excessive accumulation of fat, and this can be estimated by body mass index (BMI, calculated as body weight in kg divided by square of height in m), with the WHO classifying adults with a BMI ≥ 25 kg/m^2^ as overweight and those with a BMI ≥ 30 kg/m^2^ as obese [1]. Ischemic stroke triggers immune alterations, increasing the risk of infections. Immune system activation in the central nervous system contributes to brain tissue damage, while stroke-induced immune suppression is observed in the peripheral immune system [2, 3]. In human stroke, this poststroke immune suppression has emerged as an independent risk factor for infection when corrected for stroke severity [4, 5].

Moreover, obesity is well known to have detrimental effects on health, including an increased risk of hypertension, cardiovascular disease, and higher mortality rates [1]. There is conflicting data regarding the association between obesity and stroke outcomes. While some studies suggest better functional outcomes and mortality after stroke in obese patients [6–16], others have found no such association [17–19] or have reported different findings depending on stroke severity [19, 20] or subtype [21]. The effects of obesity on stroke patient outcomes remain controversial [22]. In human stroke patients, weight loss seems to occur during the initial hospital stay in a significant percentage of stroke patients [23] but has not been widely investigated. Moreover, stroke patients are at a high risk for cachexia in the first year after stroke [24].

Nutritional status and changes in nutritional statue are known to modulate immune responses [25]. Adipose tissue stores lipids and consists of different subtypes, such as subcutaneous and visceral adipose tissues, which have distinct functions and are considered endocrine active tissues [26]. In individuals with normal BMI, immune cells, particularly macrophages, contribute to the normal functioning of adipose tissue and have anti-inflammatory effects [27, 28]. However, in overweight and obese individuals, the accumulation of proinflammatory immune cells, their secretion of proinflammatory cytokines, and the polarization of monocytes/macrophages leads to chronic inflammation [27, 28]. Furthermore, weight loss has been demonstrated to modulate immune responses [29].

Monocytes can be divided into three different cell subpopulations by their expression of CD14 and CD16: classic monocytes (CD14^+^CD16^−^), anti-inflammatory monocytes (CD14^+^CD16^+^), and proinflammatory monocytes (CD14^dim^CD16^+^). Similarly, the granulocyte subset can be distinguished into “classic granulocytes” (CD16^+^CD62L^+^), which express high levels of CD16 and, to some degree, CD62L (a cell adhesion molecule found on leukocytes [L-selectin]); “proinflammatory” granulocytes, which are defined as CD16^dim^CD62L^+^ cells; and “anti-inflammatory” granulocytes, which are defined as CD16^+^CD62L^−^ [30, 31].

Only recently we detected reduced HLA-DR expression on monocytes and their subtypes after stroke [32]. Stroke patients showed pronounced alterations in CD32 *(Fc receptor, mediates multiple cell-type specific functions, including phagocytosis, release of inflammatory mediators, and clearance of immune complexes* [33]*)* and CD11b *(a pathogen recognition receptor, plays a critical role in pathogen recognition to initiate immune responses that are ultimately linked to the generation of adaptive immunity* [34]*)* not only in monocytes but also in granulocytes and granulocyte subpopulations [32].

The current study was designed to assess acute fat status changes post stroke and to investigate whether immune alterations (cytokines, monocytes, granulocytes) post stroke are modified by nutritional status.

## Methods

### Patient and control samples

This prospective explorative study recruited ischemic stroke patients from the dedicated stroke unit of the University Medicine Greifswald.

#### Ethics approval

The study protocol was approved by the ethics committee of the Medical Faculty, University of Greifswald (No. BB 036/17 and No. BB 050/15). Informed consent was obtained from all individual participants included in the study or through a surrogate where appropriate.

Patients admitted to the hospital for ischemic middle cerebral artery occlusion within 24 h after symptom onset were eligible for the study if the National Institutes of Health Stroke Scale (NIHSS) score was ≥ 6. Recanalization with recombinant tissue plasminogen activator and/or thrombectomy was carried out as clinically indicated. Blood samples were drawn on admission within 24 h after stroke onset (d0) as well as between 6:00 a.m. and 9:00 a.m. on the days thereafter.

To exclude confounders in the characterization of immune cells, we excluded patients with [1] clinical and laboratory signs of infection, [2] C-reactive protein (CRP) levels > 50 mg/l, and [3] current immunosuppressive medication. Age-matched control individuals were recruited from the ophthalmology clinics among patients scheduled to undergo cataract surgery. Controls had no known neurological, malignant or immunological disease.

For the analysis of soluble mediators (cytokines, chemokines, and adipokines) as well as adaptive immune cell subpopulations, 40 stroke patients and 16 control patients were analyzed on the day of admission and on days 1, 2, 3, 4, 5 and 7 thereafter. Patients were enrolled from 2015 to 2016 (Table 1).

**Table 1:** Clinical Characteristics of Cohort 1

Variable	Patient Group (N = 40)	Control Group (N = 16)
Age [Years, Mean ± SD]	71 ± 14	71 ± 8
Sex [as % female]	55%	44%
BMI [Mean ± SD]	28.45 ± 5.13	27.30 ± 3.42
First MRI Liver fat [%, Mean ± SD]	8.30% ± 2.76	13.18 ± 8.15
Second MRI Liver fat [%, Mean ± SD]	7.85% ± 2.40	NA^$^
Weight Categories		
Obese [n (%)]	15 (37.5)	3 (18.8)
Overweight [n (%)]	14 (35.0)	9 (56.3)
Normal [n (%)]	11 (27.5)	6 (15.0)
Comorbidities		
Hypertension [n (%)]	31 (77.5)	8 (50)
Diabetes mellitus [n (%)]	6 (15)	5 (31.9)
Stroke Characteristics		
Etiology		
Large-artery atherosclerosis	10 (25)	NA^$^
Cardioembolism [n (%)]	19 (47.5)	NA^$^
Stroke of other determined etiology [n (%)]	2 (5)	NA^$^
Stroke of undetermined etiology [n (%)]	9 (22.5)	NA^$^
First MRI Stroke Size [ml^3^, Median (IQR)]	31.0 (7.5–78.0)	NA^$^
Second MRI Stroke Size [ml^3^, Median (IQR)]	41.2 (8.2–89.6)	NA^$^
Initial NIHSS score [Median (IQR)]	15 (11.25-21.00)	NA^$^
Infarct size [n (%), left-sided infarcts]	20 (50.0)	NA^$^
Treatment [n (%)]	30 (75.0)	NA^$^
Systemic thrombolysis [n (%)]^&^	25 (62.5)	NA^$^
Mechanical thrombectomy [n (%)]^&^	18 (45.0)	NA^$^
Combined treatment [n (%)]	13 (32.5)	NA^$^

For the analysis of soluble mediators (cytokines, chemokines, and adipokines,) as well as adaptive immune cell subpopulations 40 stroke patients and 16 control patients were analyzed on the day of admission and on days 1, 2, 3, 4, 5 and 7 thereafter. Patients were enrolled from 2015-2016.

For the assessment of monocyte and granulocyte subpopulations, a second cohort of 17 stroke patients and 17 control patients was analyzed on admission (day 0) and at days 1, 3, and 5 thereafter. This cohort was recruited from 2017 to 2018. Body mass index was calculated using the formula BMI = weight(kg)/height(m)^2^. We used the following subgroups for further analysis: underweight BMI ≤ 18.5, normal weight BMI > 18.5 ≤ 25, overweight BMI > 25 ≤ 30, and obesity BMI > 30.

The participants’ characteristics are shown in Tables 1 and 2.

**Table 2:** Clinical Characteristics of Cohort 2

Variable	Patient Group (N = 17)	Control Group (N = 17)
Age (Years; Mean ± SD)	70 (± 12.1)	70 (± 7.2)
Sex [as % female]	6 (35.3%)	9 (52.9%)
BMI [Mean ± SD]	28.4 (± 4.1)	28.9 (5.5%)
Weight Categories		
Obese [n (%)]	6 (35.3%)	5 (29.4%)
Overweight [n (%)]	7 (41.2%)	8 (47.1%)
Normal [n (%)]	4 (23.5%)	4 (23.5%)
Comorbidities		
Hypertonia (n (%))	16 (94.1%)	12 (70.6%)
Dyslipidemia (n (%))	9 (52.9%)	9 (52.9%)
Diabetes mellitus (n (%))	3 (17.6%)	6 (35.3%)
Stroke Characteristics		
Etiology		
Large-artery atherosclerosis	3 (17.6)	NA^$^
Cardioembolism [n (%)]	9 (53.0)	NA^$^
Stroke of other determined etiology [n (%)]	2 (11.8)	NA^$^
Stroke of undetermined etiology [n (%)]	3 (52.9)	NA^$^
NIHSS score (Median (IQR))	12 (6)	NA^$^
Treatment ((n (%))^&^	15 (88.2%	NA^$^
Systemic thrombolysis (n (%))^&^	13 (76.5%)	NA^$^
Mechanical thrombolysis (n (%))^&^	7 (41.2%)	NA^$^
Combined treatment (n (%))^&^	5 (29.4%)	NA^$^
Stroke size, cm^3^ (Mean ± Std)^*^	83.3 (± 59.3)	NA^$^

For the assessment of monocyte and granulocyte subpopulations, a second cohort of 17 stroke patients and 17 control patients was analyzed on admission and on days 1, 3, and 5 thereafter. This cohort was recruited from 2017-2018. ^&^ The numbers of systemic thrombolysis procedures and mechanical thrombectomies are the total number of patients receiving the treatments and include patients receiving a combination of both. ^*^only for 8 patients MRI data for measurement of stroke size were available. ^$^NA: Not applicable. SD: standard deviation

### Infarct size (cohort 1 + 2)

Infarct size was quantified based on diffusion-weighted magnetic resonance imaging (MRI, (1.5 Tesla Magnetom Aera, Siemens) of the head and manual definition of the regions of interest (ROIs). The lesion volume was calculated semiautomatically. The patients received a brain MRI between days 1–5.

### Daily determination of fat status and weight loss over 7 days post stroke (cohort 1)

Daily, the body weight was assessed by three different methods:

First, the body weight was measured in kg with either a calibrated bed scale SECA 985 (Krauth & Timmermann, Hamburg, Deutschland) or a calibrated standing scale Soehnle Professional type 7700 (Soehnle Industrial Solutions GmbH, Backnang, Germany) depending on the mobility of the patient. Weight was determined in an undressed state or by subtracting the weight of clothes, which were weighed separately.

Second, the body fat in kg was measured by bioelectrical impedance analysis (BIA) using the B.I.A. Multi-Frequency Analyzer 2000-M (Data Input, Pöcking, Germany). BIA is a validated measurement of the composition of the body ^17^. Two electrodes were placed on the foot and the hand on the nonparetic side to determine the electrical impedance. To avoid measurement errors, the patient’s nonparetic side was selected. In the electromagnetic field, voltage drops of the flowing alternating current (I = 0.8 mA, F = 50 Hz) can be measured on the cell membranes. This results in the resistance R with information about the composition of the body fluid and the reactance Xc with information about the quality and quantity of the cells and the body cell mass. In addition, the phase angle is determined with information about the energy status of the cell. Based on these parameters plus weight and height, the fat content of the body (in%) can be determined.

Third, the skinfold thickness was determined by Harpenden Skinfold Caliper (IDASS Fitness, Glastonbury, Great Britain) in the middle of the upper arm on the nonparetic side. To guarantee comparability on days 0–7, the test point in the middle of the upper arm was marked. Measurements were taken in triplicate, and the mean skinfold thickness was calculated.

### Abdominal and liver fat (cohort 1)

Intraabdominal fat was determined by magnetic resonance imaging (MRI, (1.5 Tesla Magnetom Aera, Siemens). The patients underwent an MRI on days 1–3 and another one on days 5–8 after stroke.

A content parametric proton density fat fraction map based on chemical shift-encoded MRI (1.5 Tesla Magnetom Aera, Siemens) of the abdomen was used to quantify intraabdominal fat and liver fat^19^. The intraabdominal fat was marked on each slide. Afterward, the volume was calculated automatically by using OsiriX software (OsiriX Foundation, v. 3.8.1, 64 bit, Pixmeo Sarl, Bernex, Schwannitzerland). The hepatic fat fractions were determined in the ROI in the liver tissue (segment VII), which covered an area of 1.5–2 cm². Large vessels, visible partial volume effects, and motion artifacts were avoided when placing the ROIs.

### Determination of fat status (cohort 2)

Obesity was defined as described for cohort 1 based on body weight in kg on admission, determined by a calibrated scale.

### Definition of obesity (cohort 1 + 2)

Obesity is typically defined as the excessive accumulation of fat and can be assessed using body mass index (BMI). The World Health Organization classifies adults with a BMI ≥ 25 kg/m^2^ as overweight and those with a BMI ≥ 30 kg/m^2^ as obese.

### Blood cell counts, procalcitonin, and C-reactive protein (cohort 1 + 2)

Differential blood cell counts (XN9000, Sysmex, Norderstedt, Germany) and the concentrations of procalcitonin (PCT), and C-reactive protein (CRP) (all analyzed with the Dimension Vista, Siemens Healthcare Diagnostics, Eschborn, Germany) were quantified on days 0, 1, 2, 3, 4, 5, 7 in the central laboratory facility of the University Medicine Greifswald.

### Determination of leukocyte cell subsets (cohort 1)

The analyses of the first stroke cohort (Table 1) included evaluation of leukocytes, neutrophils, T cells, CD4 + T cells, CD8 + T cells, NK cells and B cells (anti-CD45 FITC, clone: B3821F4A; anti-CD56 PE, clone: N901/NKH-1; anti-CD16 PE, clone: 3G8, anti-CD19 ECD, clone: J3-119; anti-CD4 PE, clone: SFCI12T4D11; anti-CD8 PCD, clone: SFCI21Thy2D3; and anti-CD3_PC5, clone: UCHT1 [Beckman Coulter, Krefeld]) on days 0, 1, 2, 3, 4, 5, 7 post stroke. Cell analysis was performed on an FC500 (Beckmann Coulter, Krefeld) flow cytometer in the Department of Oncology of the University Medicine Greifswald. Cells were analysed on day of blood collection. Since all population could be clearly identified by the mentioned marker above no isotype controls were used.

### Adipokines and cytokines (cohort 1)

All patient sera were sampled on admission (day 0), day 1, day 3, and day 5 and immediately frozen at -80 °C for later analysis.

Granulocyte-macrophage colony-stimulating factor (GM-CSF), interferon (IFN)-β, IFN-γ, interleukin (IL)-1β, IL-1RA, IL-2, IL-4, IL-5, IL-6, IL-10, IL-12, IL-17, IL-17 F, IL-18, TNF-α, macrophage migration inhibitory factor (MIF), monocyte chemoattractant protein-1 (MCP-1), macrophage inflammatory protein-4 (MCP-4), MIP-3α, MIP-3β, eotaxin, soluble IL-6 receptor, E-selectin, and P-selectin were measured at the Multiplex Facility at University Leiden utilizing multianalyte profiling (xMAP) technology (Luminex, Austin, USA).

### Determination of monocyte and granulocyte subtypes (cohort 2)

The analyses of the second cohort (Table 2) included evaluation of monocyte and granulocyte subpopulations. Immune cell subpopulations were analyzed by flow cytometry after FACS staining which was performed within 1 h of blood withdrawal on day of admission and day 1, 3 and 5 post stroke (LSRII, BD Bioscience) [anti-HLA-DR Alexa Flour 488; clone: L243; anti-CD11b Brilliant Violet 421, clone: LM2; anti-CD14 PerCP/Cy5.5, clone: 63D3; anti-CD16 Brilliant Violet 650, clone: 3G8; anti-CD62Ligand PE-Cy7, clone: DREG-56; and anti-CD32-PE, clone: FUN-2 (Biolegend)]. Dead cells were excluded from analysis by the Zombie NIR TM Fixable Viability Kit (Biolegend). The results were evaluated using FlowJo Software 7.6.5 (Tree Star Inc.). The percentage of cells expressing a specific activation marker was determined as well as the amount of the specific marker per cell as defined by the mean fluorescence intensity (MFI). Fluorescence minus one controls (FMO) were used to distinguish different monocyte and granulocyte populations.

### Statistical analysis

Statistical analyses were performed by R version 4.1.0 [35]. with the packages emmeans, tidyverse, lmerTest, car, ggeffects and bestNormalize. The datasets that were not Gaussian distributed were transformed by orderNorm using the bestNormalize package. Correlations were determined by Spearman analysis.

Additionally, multiple regression analysis was calculated to evaluate the influence of BMI on all measured cell parameters independent of sex, age, therapy, day after stroke and stroke volume.

A generalized linear mixed model for a gamma distribution with log-link function was used to evaluate the influence of BMI on immune alterations over time adjusted for the baseline covariables sex, age, therapy, day after stroke and stroke volume. Patient ID was included as a random factor. BMI/day interaction factor was included to check whether the progression over time was BMI dependent. Continuous covariables were z-transformed. For better visualization, we retransformed β-coefficients to percentage change/1 standard deviation BMI. Therefore, the figures represent significant alterations over the study period, whereby the three figure panels represent the results for BMI values corresponding to -1 SD, the mean, and + 1 SD. A p value < 0.05 was regarded as significant.

## Results

### Weight loss during the acute phase of stroke occurs in normal and underweight patients (cohort 1)

**Fig. 1:**
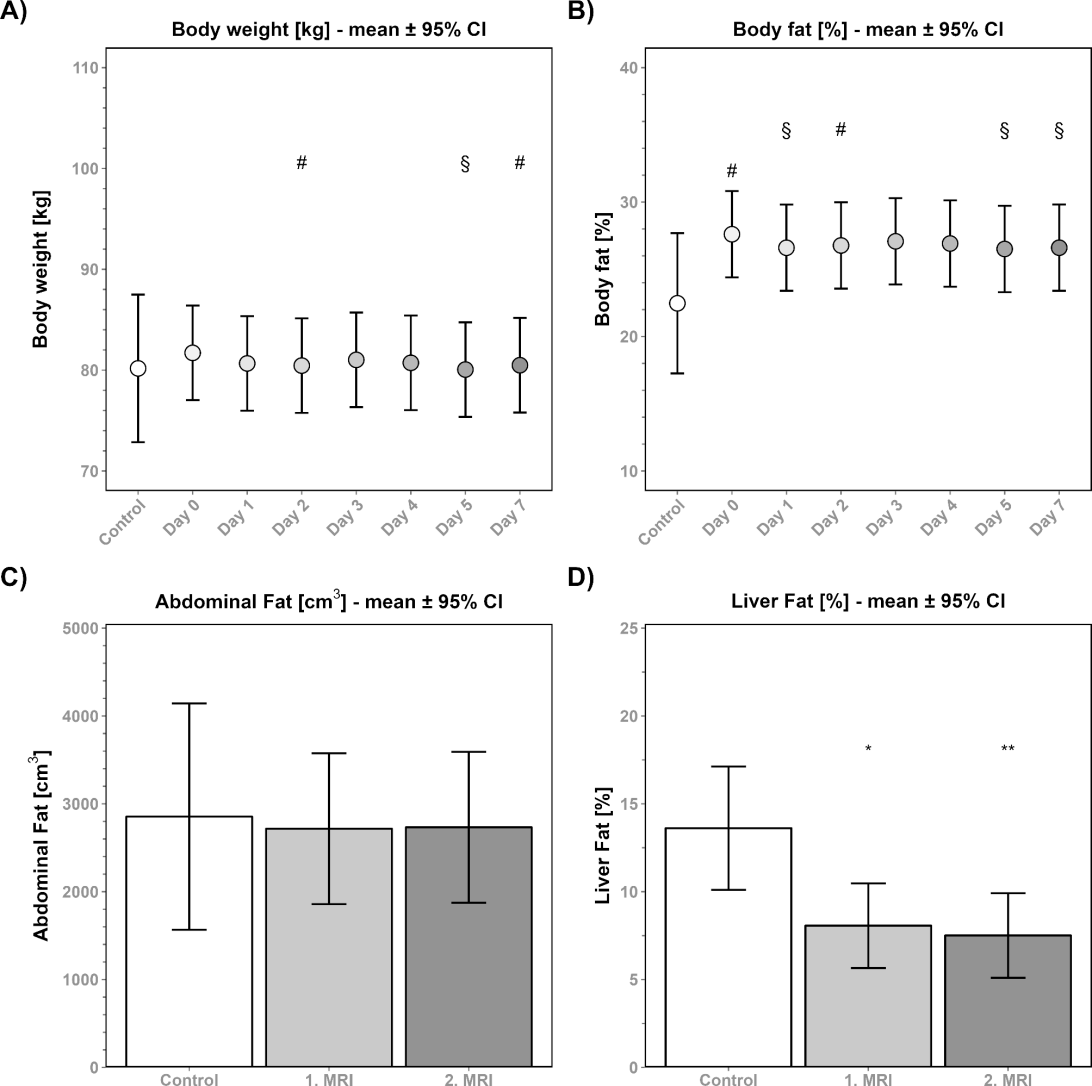
Body weight, body fat, abdominal fat and liver fat in stroke patients Stroke patients were analyzed regarding body weight (**A**), body fat (**B**), abdominal fat (**C**) and liver fat (**D**). Body weight was measured by a calibrated bed scale or calibrated standing scale depending on the mobility of the patient on the day of admission (day 0) and on days 1, 2, 3, 4, 5 and 7 after stroke (n_d0−d7_ = 40). Weight was measured in an undressed state or by measuring clothes separately. To measure the infarct size, intraabdominal fat MRI was used (3.0 Magnetic Resonance Scanner). Controls received one MRI, while patients received one on day 1 and one on days 3–5 (n_Control_ = 8, n_MRT1_ = 17, n_MRT2_=17). *p < 0.05; **p < 0.01 in comparison to controls; ^§^p < 0.05 in comparison to stroke patients on the day of admission; mean ± 95% confidence interval

On admission, body weight of stroke patients was 81.18 ± 14.93 kg (mean ± SD), and weight of control patients was 80.18 ± 16.16 kg. On day 5 after stroke onset, body weight of stroke patients was reduced compared to admission (Fig. 1A). Similar differences were seen for the percentage of body fat of stroke patients on days 1, 5 and 7 (Fig. 1B). Abdominal fat and liver fat were not altered between the first MRI and the second MRI. While abdominal fat volume was similar in healthy controls and stroke patients (Fig. 1C), Liver fat was reduced in stroke patients at both MRI measurements compared to controls (Fig. 1D). Skinfold thickness was altered only on day 7 compared to admission (mean ± SD; d0: 21.32 mm ± 10.65 mm; d7: 21.60 mm ± 8.982 mm) (data not shown).

**Fig. 2:**
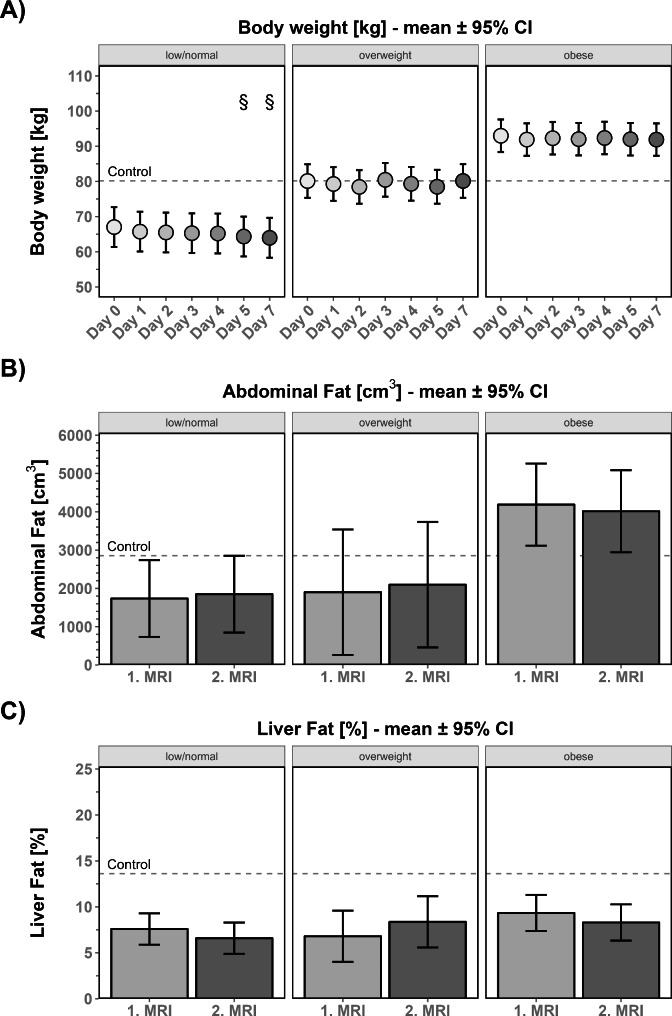
Fat status within different BMI groups To analyze the effect of adiposity, the cohort was divided into three subgroups with a BMI below 25 kg/m² (white bars, normal and underweight, 12 patients), between 25 and 30 kg/m² (bright gray bars, overweight, 14 patients) and above 30 kg/m² (dark gray bars, obese, 14 patients). In (**A**), body weight, which was measured by calibrated scales, is shown for these 3 BMI groups. Abdominal fat (**B**) and liver fat (below 25 kg/m²: n_MRT1_ = 8, n_MRT2_= 8; between 25 and 30 kg/m²: n_MRT1_ = 3, n_MRT2_= 3; above 30 kg/m²:: n_MRT1_ = 7, n_MRT2_= 7) (**C**) were assessed by 2 MRIs (white bars = normal and underweight patients; bright gray bars = overweight patients; dark gray = obese patients) ^§^p < 0.05 in comparison to stroke patients on the day of admission, mean ± 95% confidence interval

To analyze the effect of adiposity, the cohort was divided into three subgroups by BMI: below 25 kg/m² (normal and underweight, 12 patients), between 25 and 30 kg/m² (overweight, 14 patients) and above 30 kg/m² (obese, 14 patients). In the below 25 kg/m² BMI group, weight loss was significant when comparing the weight on days 5 and 7 to the weight on admission. In all other BMI groups, we detected no significant changes in body weight throughout the observation period compared to admission (Fig. 2A). In addition, no changes were detected for body fat or skinfold thickness (data not shown).

In the subgroup below 25 kg/m², abdominal fat was 1534 cm³ ± 1448 cm³, and at the second MRI, it was 1609 cm³ ± 1268 cm³ (mean ± SD; between 25 and 30 kg/m²: MRI 1: 3631 cm³ ± 2513 cm³, MRI 2: 3828 cm³ ± 2633 cm³; above 30 kg/m²: MRI 1: 3707 cm³ ± 1389 cm³, MRI 2: 3544 cm³ ±: 1034 cm³). Neither abdominal fat nor liver fat (mean ± SD, below 25 kg/m²: MRI 1: 8.65% ± 3.863%; MRI 2: 7.213% ± 1.97%; between 25 and 30 kg/m²: MRI 1: 6.8% ± 3.081%, MRI 2: 8.367% ± 4.332%; above 30 kg/m²: MRI 1: 9.529% ± 2.156%; MRI 2: 8.557% ± 1.88%) (Fig. 2B, C) differed between the MRI at admission and the second MRI independent of the BMI group.

### Stroke induces loss of lymphocyte subsets and increase in neutrophils (cohort 1)

In stroke patients of cohort 1, we observed the well described immunological changes induced by acute stroke with a decrease in T-helper lymphocytes and an increase in neutrophils in peripheral blood. Details are shown in supplemental figures I and II.

### BMI correlates with adipokines and inflammatory markers of poststroke immune suppression (cohort 1)

Leptin (p value = 1.047e-07; r = 0.4164) and adiponectin (p value = 0.00144; r -0.2579) significantly correlated with BMI.

Multiple regression analysis was performed to evaluate the influence of BMI on all investigated cellular parameters, cytokines, chemokines and adipokines independent of sex, age, stroke treatment, days after stroke and stroke volume.

**Fig. 3:**
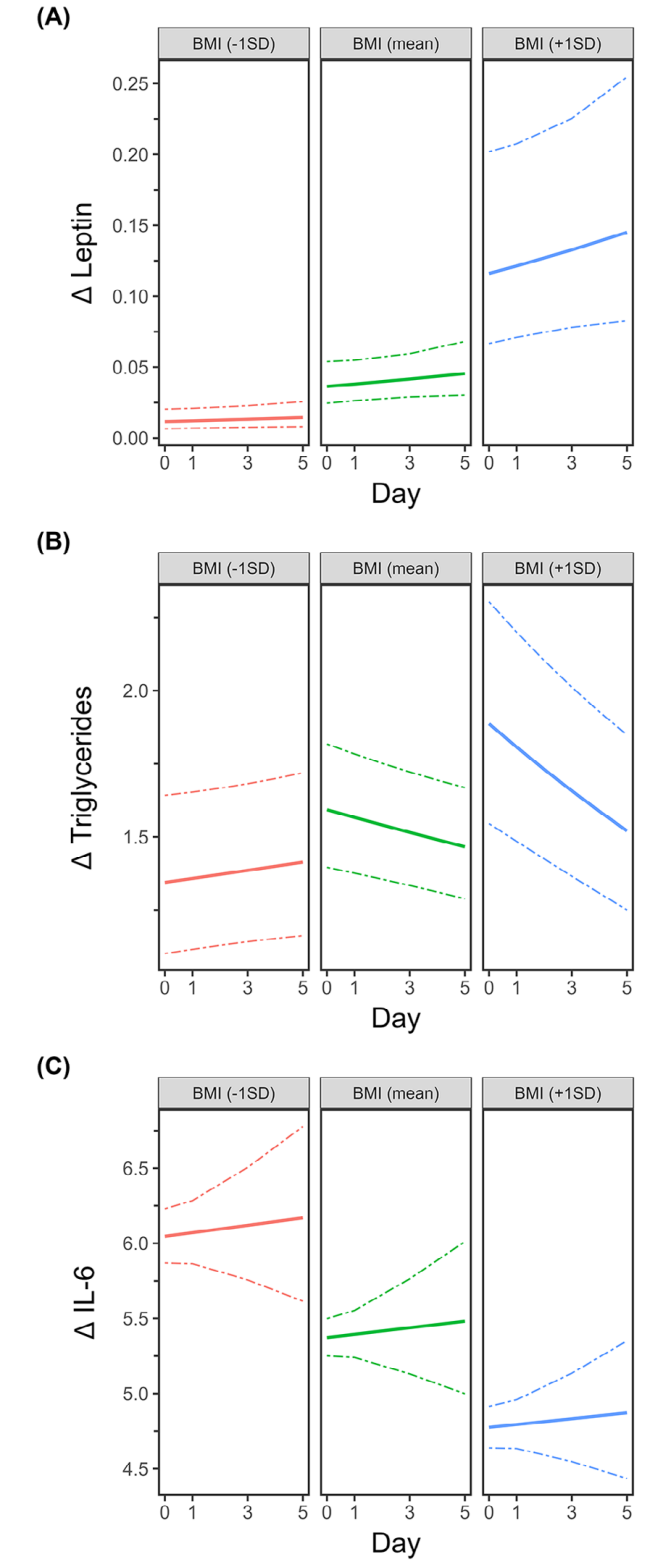
Higher BMI ratios are associated with higher levels of leptin and triglycerides but reduced IL-6 levels after stroke Stroke patients were analyzed and compared to healthy controls on the day of admission (day 0) and on days 1, 3 and 5. Multiple regression analysis was performed to evaluate the influence of BMI on leptin, triglyceride and IL-6 levels independent of sex, age, stroke treatment, days after stroke and stroke volume. BMI ratios: For better visualization, we retransformed β-coefficients to percentage change/1 standard deviation BMI. Therefore, the figures represent significant alterations over the study period, whereby the three figure panels represent the results for BMI values corresponding to -1 SD, the mean, and + 1 SD

BMI was positively associated with leptin (p < 0.0001; β-coefficient = 1.16014) and overall triglyceride concentrations (BMI: p = 0.02605, β-coefficient = 0.16584). (Fig. 3A, B)

**Fig. 4:**
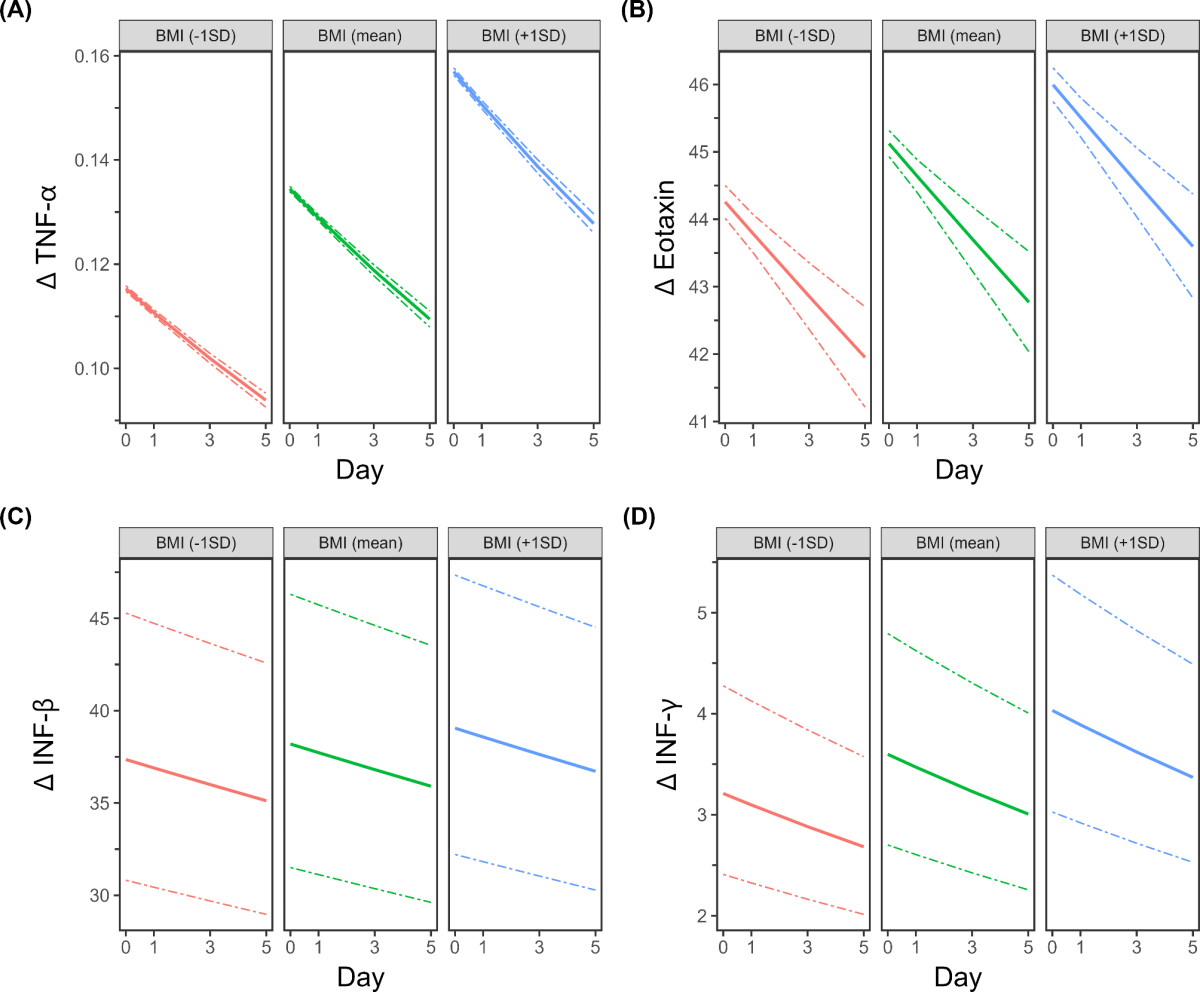
Higher BMI ratios are associated with higher levels of TNF-α, eotaxin, IFN-β and IFN-γ after stroke Stroke patients were analyzed and compared to healthy controls on the day of admission (day 0) and days 1, 3 and 5. Multiple regression analysis was performed to evaluate the influence of BMI on TNF-α, eotaxin, IFN-β and IFN-γ levels independent of sex, age, stroke treatment, days after stroke and stroke volume. BMI ratios: For better visualization, we retransformed β-coefficients to percentage change/1 standard deviation BMI. Therefore, the figures represent significant alterations over the study period, whereby the three figure panels represent the results for BMI values corresponding to -1 SD, the mean, and + 1 SD

While IL-6 was negatively associated with BMI values (p < 0.0001, β-coefficient = -0.120484) (Fig. 3C), eotaxin, IFN-β, IFN -γ and TNF-α were upregulated with increasing BMI (eotaxin: p < 0.0001, β-coefficient = 0,019109; IFN -β p < 0.0001, β-coefficient = 0,022613; IFN -γ: p < 0.0001, β-coefficient = 0,116371; TNF-α: p < 0.0001, β-coefficient = 0,156474) (Fig. 4A-D). All other parameters did not correlate with BMI.

### Proinflammatory monocyte and granulocyte subpopulations increase with BMI (cohort 2)

**Fig. 5:**
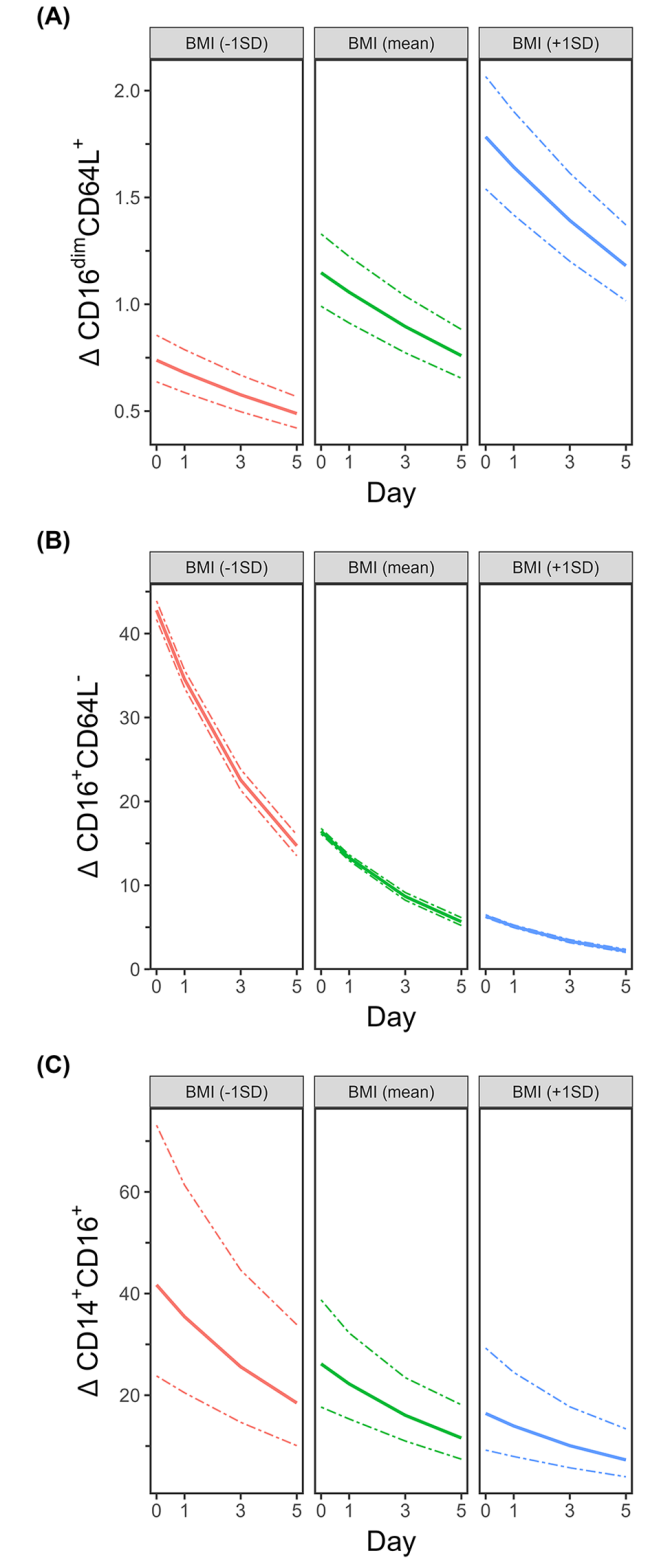
Higher BMI ratios are associated with CD16^dim^CD62L^−^ granulocytes but with reduced levels of CD16^+^CD62L^−^ granulocytes and CD14^+^CD16^+^ monocytes Stroke patients were analyzed and compared to healthy controls on the day of admission (day 0) and on days 1, 3, and 5. Multiple regression analysis was performed to evaluate the influence of BMI on CD16^dim^CD62L^−^ and CD16^+^CD62L^−^ granulocytes as well as on CD14^+^CD16^+^ monocytes independent of sex, age, stroke treatment, days after stroke and stroke volume. BMI ratios: For better visualization, we retransformed β-coefficients to percentage change/1 standard deviation BMI. Therefore, the figures represent significant alterations over the study period, whereby the three figure panels represent the results for BMI values corresponding to -1 SD, the mean, and + 1 SD

Analysis was performed as described above. The number of proinflammatory CD16^dim^CD62L^+^ granulocytes correlated positively with BMI (p < 0.0001; β-coefficient = 0.441009), while the number of CD16^+^CD62L^−^ granulocytes was negatively associated with BMI (p < 0.0001; β-coefficient = -0.956042) (Fig. 5A, B). The number of anti-inflammatory CD14^+^CD16^+^ monocytes was reduced in patients with higher BMI values (p = 0.0235, β-coefficient = − 0.46632) (Fig. 5C). All other parameters did not correlate with BMI.

## Discussion

In this study, we examined changes in body weight, immune cell fractions, and levels of cytokines, chemokines, and adipokines after stroke and their correlation with patient BMI. Our goal was to understand the impact of obesity on immunosuppression following a stroke. We observed an average weight loss of 1.7 ± 0.5 kg in our stroke cohort within the first seven days, primarily occurring between admission and day one. Various factors, such as restricted food access due to planned medical interventions and stroke-related dysphagia, may contribute to this weight loss. Additionally, while weight on days one through seven was gathered at a similar time point in the morning, admission weights were obtained at the given time of admission throughout the day. To avoid microtrauma that could result in bleeding, transurethral catheterization is not carried out prior to thrombolysis. Therefore, even an estimation of such bias is impossible.

A previous study reported that 9% of stroke patients experienced significant weight loss, which was associated with a poor prognosis [23]. By applying similar criteria, we found that 45% of our patients had significant weight loss on day 5 or 7 compared to their admission weight. The higher rate of weight loss in our study may be due to the fact that our patients had more severe strokes. However, we did not investigate whether the patients’ BMI at admission influenced their weight loss. We also performed assessments of body composition, skinfold thickness, and fat measurements, which did not show any significant changes indicating stroke-induced breakdown of fat tissue. Since the overall weight loss was small and not reflected in the other parameters we did not investigate the effect of changes in nutritional status on the post stroke immune response. Our data highlight the metabolic differences post stroke in humans compared to rodent stroke models in which weight loss following stroke is severe [36]. This needs to be considered, when experimental stroke models apply overweight animals to elucidate metabolic effects on stroke outcome.

Our findings confirm previously observed immunological changes in the immediate aftermath of a stroke, such as increased granulocyte counts, reduced numbers of CD4+-T-lymphocytes, and elevated levels of acute phase proteins [4, 37, 38]. However, some of these changes were only marginal in our patients. We observed a trend toward increased leukocyte and decreased B-cell counts early after stroke. However, total lymphocyte, CD8+-T-lymphocyte and NK cell counts did not differ from control values. Another study [39] reported no or minor changes in leukocyte and lymphocyte counts. Infarct volume was discussed as a relevant parameter determining the immunological changes, but our median stroke volume of 31 cm^3^ was well within the range of previously reported trials, similar to Hug et al. [39]. Our stroke patients had higher levels of C-reactive protein (CRP) compared to controls, which indicates an acute phase response to brain injury and is consistent with previous research that found a correlation between CRP levels and infarct size [40, 41].

Leptin levels peaked modestly on day three after the stroke, which is in line with previous studies that found increased leptin levels in etiological subgroups of stroke patients [42, 43]. The time courses of leptin [44] and adiponectin [45] in our study were similar to those previously reported. Stroke patients with metabolic syndrome had increased plasma levels of the proinflammatory cytokines CRP, IL-6 and TNF-α but decreased levels of the anti-inflammatory cytokine IL-10 compared to stroke patients without metabolic syndrome and nonstroke patients [46]. Our data also showed higher levels of IFN-β, IFN-γ, and TNF-α after stroke, regardless of factors such as age, sex, time of treatment, and stroke volume.

Additionally, data regarding eotaxin are in line with the findings of Roy-O’Reilly et al. [47]. Herein, the eotaxin level predicts long-term functional outcomes in patients following ischemic stroke.

Triglyceride and leptin levels as well as IFN-β, IFN-γ and TNF-α were elevated with higher BMI values, and the IL-6 concentration was reduced with higher BMI values after stroke in our data. This is in contrast to several obesity-related cytokine studies [48]. One possible explanation for the different results between Tutololmondo et al. [46] and our results is that we compared only patient groups in different BMI ranges, while the aforementioned authors compared patients with and without metabolic syndrome. Inflammatory mediator levels are similarly elevated in patients suffering from many comorbidities of obesity independent of body weight, including atherosclerosis, diabetes and steatohepatitis [49, 50]. A state of chronic systemic inflammation may therefore underlie the pathogenesis of many obesity-related comorbidities. Furthermore, different sources of aberrant serum cytokine levels in obesity might be relevant, even for stroke. Since the transcript levels of IL-6 in the PBMCs of obese patients were low, leukopenia after stroke might induce lower serum IL-6 levels in obese patients than in lean patients [51].

Another explanation is that our cohort of obese patients was only moderately obese, with a median BMI of 32 kg/m^2^, and at least some proinflammatory markers correlated with high BMI values [52], implying that the effects of obesity on poststroke immunological changes can be seen with higher BMI values.

Although we found typical cellular poststroke alterations, only granulocyte and monocyte subsets were regulated via BMI. Herein, the numbers of anti-inflammatory CD14^+^CD16^+^ monocytes and CD16^+^CD62L^−^ granulocytes were reduced in patients with higher BMI values, while that of proinflammatory CD16^dim^CD62L^+^ granulocytes was increased. A recent study conducted in 622 healthy volunteers showed that proinflammatory CD14^dim^CD16^+^ monocytes were correlated with BMI [53]. Additionally, another study confirmed a shift toward proinflammation for obesity within a subpopulation of monocytes [54].

We are aware that our study has several limitations. The number of patients and controls is comparable to other studies [5, 35, 36, 47] but might be insufficient to detect small but relevant effects due to limited statistical power. Due to logistic restraints, the study protocol did not include cerebral and abdominal MRI data before stroke treatment and therefore might have missed relevant acute effects. Furthermore, one principal limitation of our study is that the immunological status of our patients before the stroke was unknown, and therefore, part of the observed effects might be preexisting and unrelated to stroke. This is especially true for values that remained stable throughout the course of our trial. Due to our small study size, subtle mismatches in patient and control characteristics might exist, despite our effort to match patients and controls. As stated above, admission weights were measured at any time in the day due to the emergency nature of most encounters.

On admission 11 (27.5%, cohort 1) and 5 (29,4%, cohort 2) patients were treated with HMG-CoA reductase inhibitors, after day 1 all patients received either atorvastatin 40-80 mg or simvastatin 40 mg daily. Triglyceride values among stroke patients and control subjects were similar on admission but in stroke patients´ serum values decreased slightly. Secondary prophylactic administration of statins after ischemic stroke [55]reduces free fatty acids in blood plasma and thus might interfere with our results.

### Summary

In this exploratory study we observed both obesity- and stroke-induced proinflammatory shifts for several immune parameters. Whether this proinflammation influences stroke outcome remains speculative. The obesity paradox describes the unexpected, improved prognosis and lower mortality rates found in several diseases in patients with higher body weight. Only few clinical studies have assessed the obesity paradox in stroke, showing contradictory results. A recent study assessing the functional outcome of obese vs. nonobese stroke patients at 3 months found no differences (p = 0.882). Proinflammatory IL-6 levels (p = 0.002) were differentially regulated. Initial IL-6 levels were higher in obese patients than in nonobese patients but decreased over the first week in obese patients, while in nonobese patients, IL-6 levels increased [56]. Therefore, future studies are needed to delineate obesity-triggered inflammation and evaluate its clinical effects on stroke patients.

## Electronic supplementary material

Below is the link to the electronic supplementary material.

Supplementary Material 1

Supplementary Material 2

## Data Availability

The datasets acquired and/or analyzed during the current study and the R script are available from the corresponding author upon reasonable request.
