# Supplementary figures and images for: Effects of body mass index on the immune response within the first days after major stroke in humans
Source: Neurol Res Pract. 2023 Aug 17;5:42. doi: 10.1186/s42466-023-00269-1 (PMC10433619; doi:10.1186/s42466-023-00269-1)

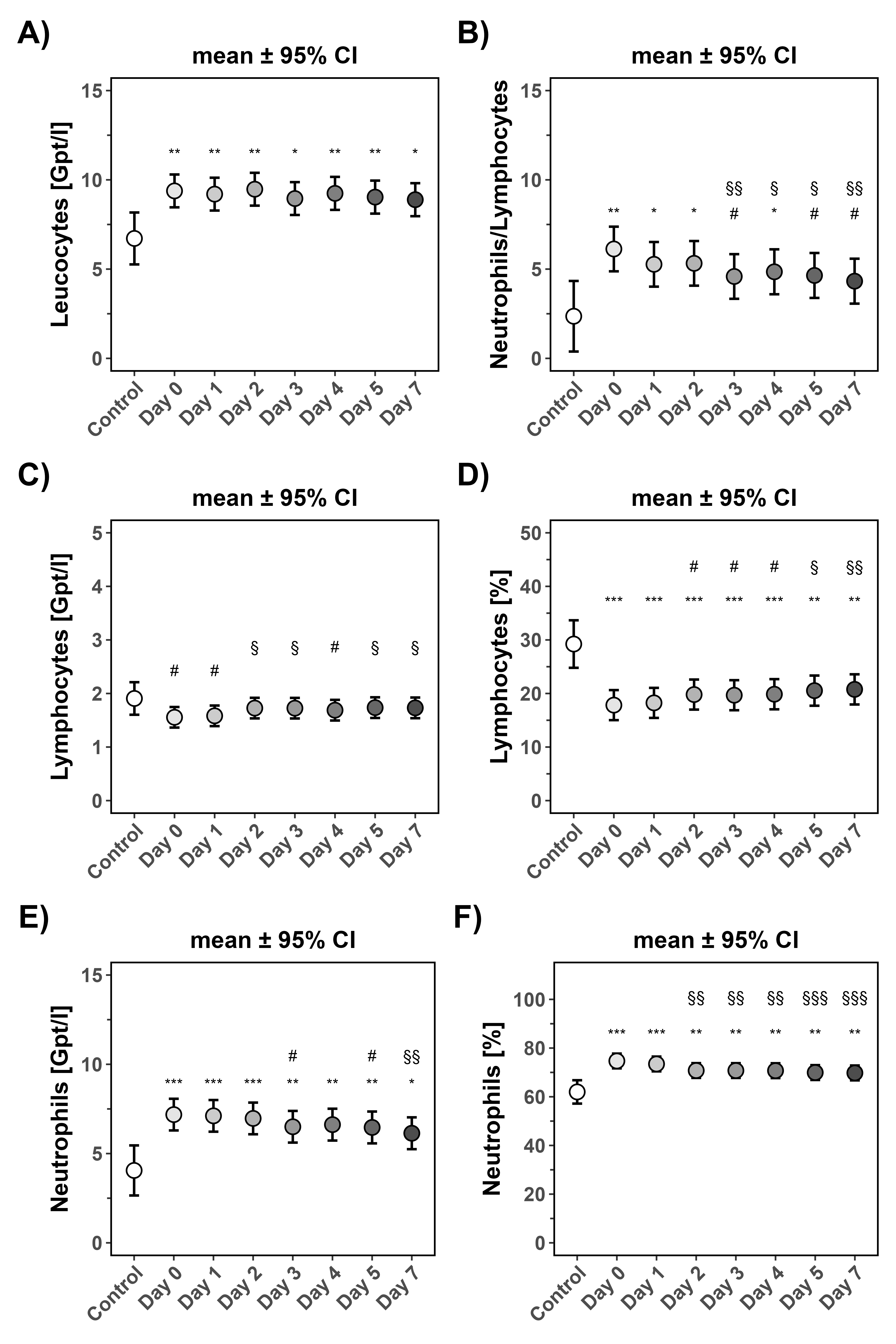

Supplement: Supplementary file 1 — Supplementary Material 1 [file 42466_2023_269_MOESM1_ESM.png]

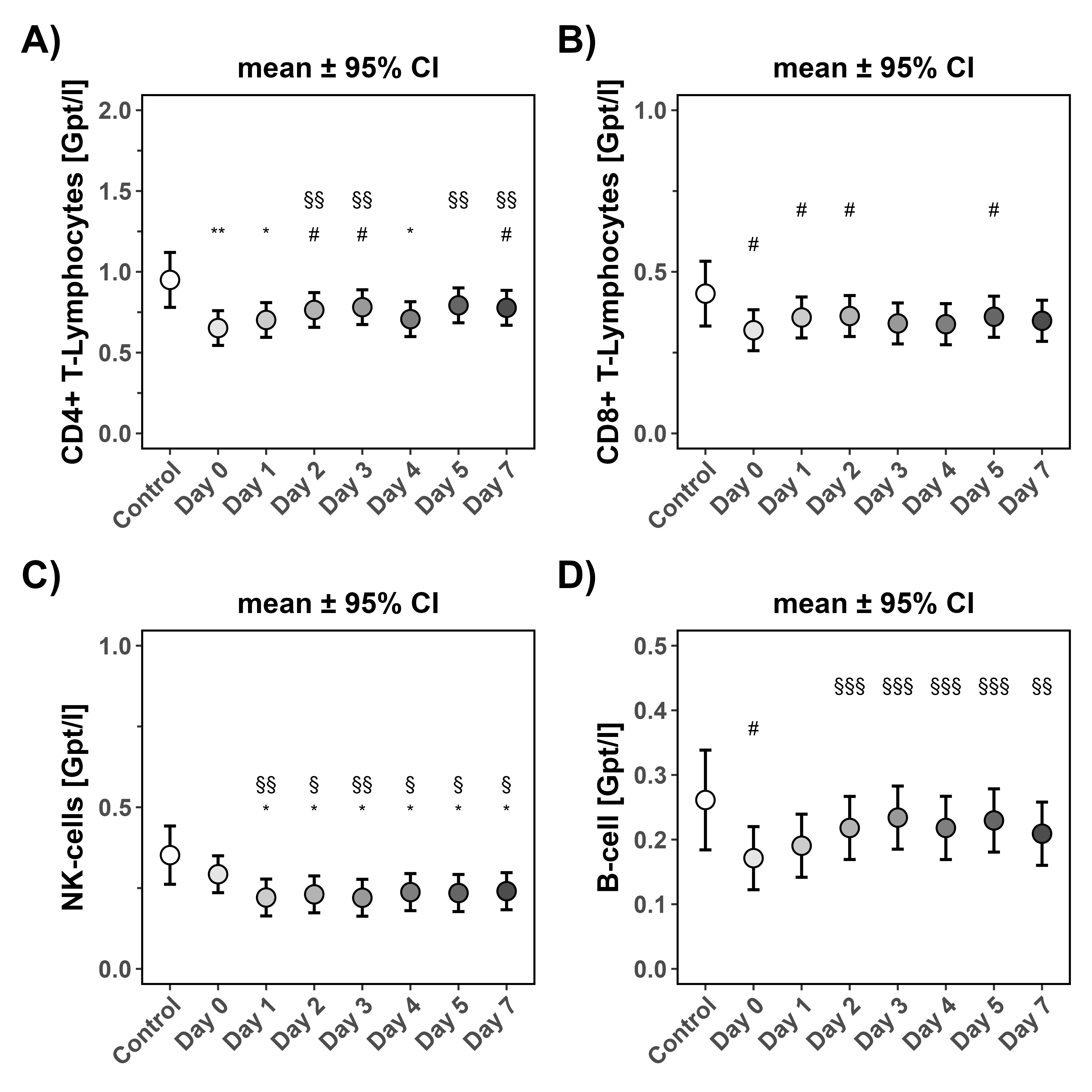

Supplement: Supplementary file 2 — Supplementary Material 2 [file 42466_2023_269_MOESM2_ESM.png]
